# Roles of Two *Sox9* Genes during Gonadal Development in Japanese Flounder: Sex Differentiation, Spermatogenesis and Gonadal Function Maintenance

**DOI:** 10.3390/ijms19020512

**Published:** 2018-02-08

**Authors:** Xiaojing Li, Haiyang Yu, Yujue Wang, Xiaobing Liu, Yuezhong Liu, Jiangbo Qu, Xubo Wang

**Affiliations:** Ministry of Education Key Laboratory of Marine Genetics and Breeding, College of Marine Life Sciences, Ocean University of China, Qingdao 266003, China; lixiaojing920825@163.com (X.L.); yuhaiyangyx@163.com (H.Y.); wangyujue1236@126.com (Y.W.); liuxiaobing132@163.com (X.L.); yuezhong_liu@163.com (Y.L.); jbq0315@163.com (J.Q.)

**Keywords:** Japanese flounder, *Posox9a* and *Posox9b*, dimorphic expression, sex differentiation, 17α-Methyltestosterone administration, gonadal development

## Abstract

The transcription factor *sox9* has been implicated in cartilage formation and testis determination in mammals. Here, two duplicates of *sox9* were found in Japanese flounder (*Paralichthys olivaceus*) named *Posox9a* and *Posox9b*, respectively. Phylogenetic and gene structure analyses revealed that *Posox9a* and *Posox9b* were homologous to that of teleosts and tetrapods. Quantitative real-time polymerase chain reaction (qRT-PCR) showed that both *Posox9a* and *Posox9b* expressed higher in testis than in ovary of adult tissues. The in situ hybridization (ISH) analysis of gonads showed that *Posox9a* and *Posox9b* mRNA were both detected in oocytes, Sertoli cells and spermatocytes. During sex differentiation, the expression of *Posox9a* exhibited obvious sexual dimorphic expression from 60 days after hatch (dah) with higher expression in male preferred individuals than female preferred individuals and increased gradually from 30 to 100 dah. A similar pattern was detected in *Posox9b* expression. After injection of androgen (17α-methyltestosterone) of different concentrations, the expression level of *Posox9b* increased significantly, whereas *Posox9a* did not change obviously. These results indicated that the two *sox9* genes of Japanese flounder had converse functions in sex differentiation, whereas their differences in 17α-methyltestosterone administration were obvious and worthwhile for exploring evolutionary and adaptive significance. This study provided a foundation for further exploration of the roles of *Posox9* genes during the sex determination and differentiation, spermatogenesis and gonadal function maintenance of Japanese flounder.

## 1. Introduction

Research on the *Sox* gene family began with the seminal discovery of the mammalian testis-determining factor—SRY [1,2]. The identification and homology-based analysis of the high mobility group (HMG), DNA-binding domain of SRY led to the discovery of the Sry-related HMG box (SOX) transcription factor family [3]. In general, proteins containing an HMG domain with 60% or higher amino acid similarity to the HMG domain of SRY are referred as Sox proteins [4]. Sox proteins that share an HMG domain with more than 80% sequence identity are divided into different HMG groups termed A to H [5]. 

*Sox9* gene, a member of SoxE subfamily, is a transcription factor required for cartilage formation and testis determination in mammals [6,7]. SRY initiates a cascade of gene networks through the direct regulation of *Sox9* expression and promotes supporting cell differentiation, Leydig cell specification, vasculature formation and testis cord development [8]. Mutations in the human *SOX9* gene cause skeletal defects and male-to-female sex reversal, indicating its essential roles in chondrogenesis and testis development. In mice and chickens, the expression of *Sox9* is down-regulated in the differentiating gonad just before ovary formation, but its expression in Sertoli cells of the developing testis persists throughout adulthood [9,10,11]. Two *sox9* genes have been found in several teleost species, such as zebrafish (*Danio rerio*), fugu (*Takifugu rubripes*), stickleback (*Gasterosteus aculeatus*), rice field eel (*Monopterus albus*) and rainbow trout (*Oncorhynchus mykiss*). Zebrafish *sox9a* transcripts have been detected in Sertoli cells of the testis, while *sox9b* has been detected in ovaries [12]. Fugu *sox9a* expresses at higher levels in the testis than in the ovary, whereas its *sox9b* is only detected in the ovary [13]. In rice field eel, both *sox9a1* and s*ox9a2* express in the testis, ovary, as well as in the ovotestis of intersex individuals. In sablefish (*Anoplopoma fimbria*), *sox9* mRNA prominently expresses in the testis, which indicates that *sox9* is related to the formation and differentiation of the testis [14]. This male-favored expression profile at the stage of testis formation is also observed in freshwater and marine turtles [15], denoting that the functional importance of *sox9* for male differentiation is conserved among tetrapods. Furthermore, with the ablation of *Sox9*, mice exhibit defects in the specification of oligodendrocytes and astrocytes, suggesting important roles of *sox9* in the development of central nervous system [16]. In *Xenopus*, the depletion of SOX9 protein in developing embryos causes a dramatic loss of neural crest progenitors and an expansion of the neural plate, indicating that SOX9 is required for cranial neural crest development [17]. All of these findings implied that *sox9* is a multifunctional gene, especially in the differentiation and maintenance of the testis and the development of the central nervous system. 

Because of the whole genome duplication event, two *sox9* duplicates (named *Posox9a* and *Posox9b*) are found in Japanese flounder (*Paralichthys olivaceus*), which is one of the most important species in aquaculture with a stable XX/XY sex determination system [18]. In this study, we focus mainly on the molecular and genetic features of the two *sox9* genes of Japanese flounder from analyses of larvae and adults in sex development, as well as the effects of androgen (17α-methyltestosterone) administration on their expression. The results will improve the current understanding of the expression patterns, biological functions and evolutionary significance of Japanese flounder *sox9* genes in sex differentiation, gonadal development and maintenance. 

## 2. Results

### 2.1. Molecular Characterization of Posox9a and Posox9b

#### 2.1.1. Cloning and Sequence Analysis of Posox9a and Posox9b

**Figure 1:**
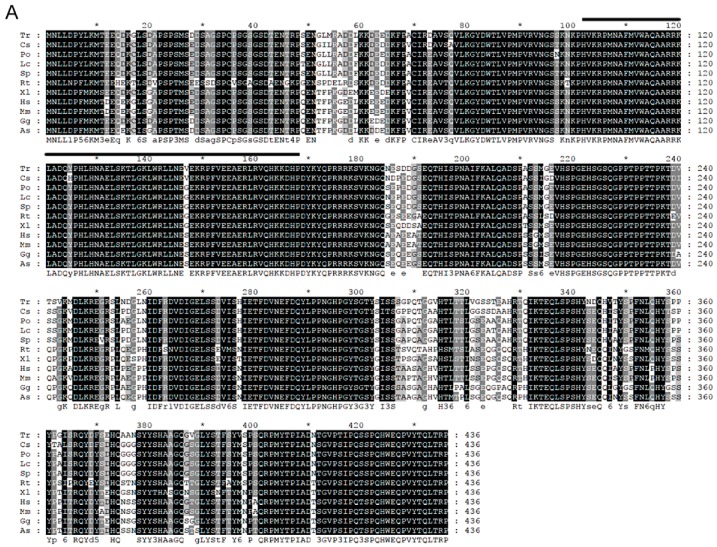

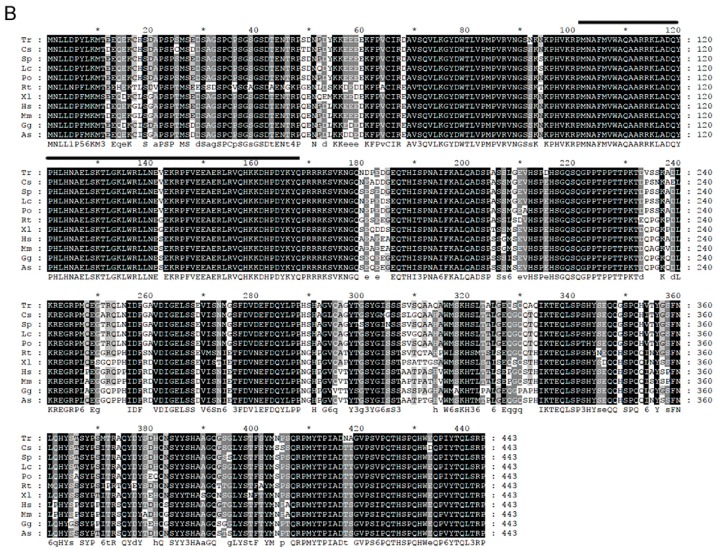
Multiple alignment of Sox9 amino acid sequences in different species. (**A**) PoSox9a; (**B**) PoSox9b. The black shadow region indicates positions where all sequences selected share the same amino acid residue. Identical amino acids are in dark grey background. The conserved regions are indicated with black lines. GenBank accession numbers of these sequences are as follows: *Takifugu rubripes* Sox9a (Tr, AAQ18508.1); *Cynoglossus semilaevis* Sox9a (Cs, XP_008313399.1); *Stegastes partitus* Sox9a (Sp, XP_008295125.1); *Lates calcarifer* Sox9a (Lc, XP_018541051.1); *Paralichthys olivaceus* Sox9a (Po, unpublished); *Rhincodon typus* Sox9a (Rt, XP_020382797.1); *Xenopus laevis* Sox9 (Xl, NP_001087942.1); *Homo sapiens* SOX9 (Hs, NP_000337.1); *Mus musculus* Sox9 (Mm, NP_035578.3); *Gallus gallus* Sox9 (Gg, NP_989612.1); Alligator sinensis Sox9 (As, XP_006029531.1); *Takifugu rubripes* Sox9b (Tr, AAL32172.1); *Cynoglossus semilaevis* Sox9b (Cs, XP_016895678.1); *Stegastes partitus* Sox9b (XP_008301579.1); *Lates calcarifer* Sox9b (Lc, XP_018541051.1); *Paralichthys olivaceus* Sox9b (Po, ACO40490.1); *Rhincodon typus* Sox9b (Rt, XP_020382797.1).

Sequences of *Posox9a* and *Posox9b* were obtained from the genome and transcriptome of Japanese flounder by the local alignment search tool (BLAST+ 2.6.0). Besides, the full-length coding region of the two sequences was confirmed by amplifying, cloning, and sequencing and they were significantly similar to other *sox9a* and *sox9b* genes by BLAST search at National Center of Biotechnology Information (NCBI), respectively. Comparison of genomic and cDNA sequences showed that both *Posox9a* and *Posox9b* contained three exons and two introns. The predicted amino acid sequences of PoSox9a and PoSox9b were 497 and 478 residues respectively. Based on bioinformatics analysis, highly conserved HMG-box domains of the Sox superfamily was found in PoSox9a and PoSox9b sequences from residue 103–169 and 102–169, respectively (Figure 1).

#### 2.1.2. Homology and Phylogenetic Analysis

**Figure 2:**
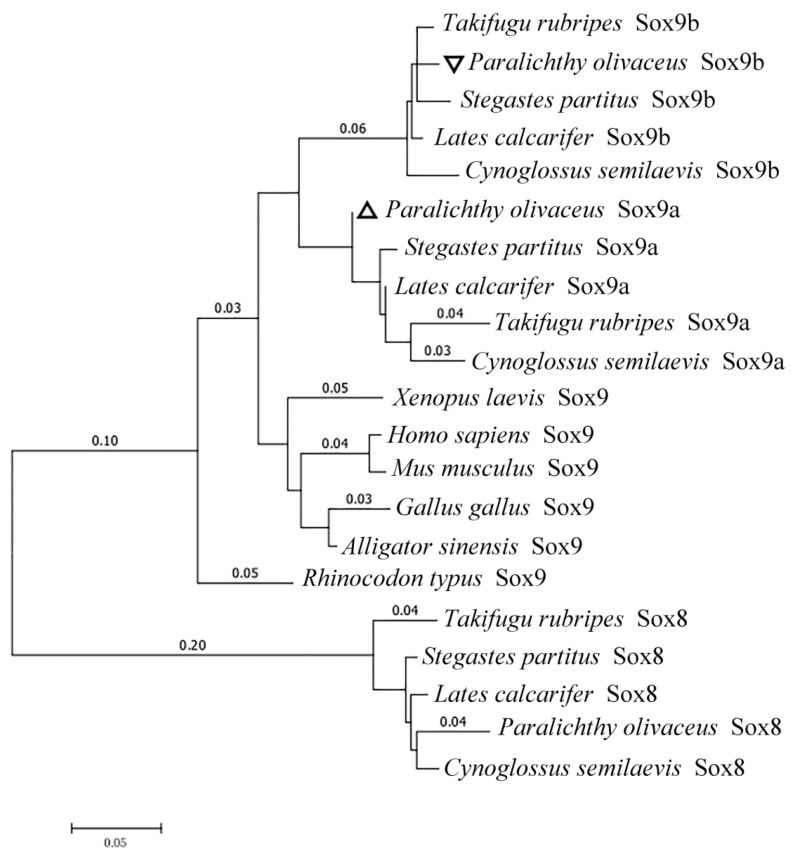
Phylogenetic tree of PoSox9a and PoSox9b in comparison with Sox9 and Sox8 proteins in other representative vertebrates using predicted amino acid sequences. The two triangular symbols in the picture indicate the two Sox9 in *Paralichthy olivaceus*. The phylogenic tree was generated by MEGA6.0 with the WAG model. The scale bar is 0.05. The GenBank accession numbers are as follows: *Homo sapiens* Sox9, NP_000337.1; *Mus musculus* Sox9, NP_035578.3; *Gallus gallus* Sox9, NP_989612.1; *Alligator sinensis* Sox9, XP_006029531.1; *Xenopus laevis* Sox9, NP_001087942.1; *Rhincodon typus* Sox9, XP_020382797.1; *Lates calarifer* Sox9a, XP_018541051.1; *Stegastes partitus* Sox9a, XP_0082905125.1; *Paralichthys olivaceus* Sox9a, unpublished; *Cynoglossus semilaevis* Sox9a, XP_008313399.1; *Takifugu rubripes* Sox9a, AAQ18508.1; *Cynoglossus semilaevis* Sox9b, XP_016895678.1; *Stegastes partitus* Sox9b, XP_008301579.1; *Lates calarifer* Sox9b, XP_018536323.1; *Takifugu rubripes* Sox9b, AAL32172.1; *Paralichthys olivaceus* Sox9b, ACO40490.1; *Lates calarifer* Sox8, AKI32583.1; *Stegastes partitus* Sox8, XP_008301648.1; *Cynoglossus semilaevis* Sox8, XP_008328025.1; *Takifugu rubripes* Sox8, NP_001072112.1.

To evaluate the evolutionary relationship between the predicted PoSox9a/b and another vertebrate group E Sox, a phylogenetic tree was generated using a maximum likelihood algorithm by MEGA6.0 with a Whelan and Goldman (WAG) model based on the amino acid sequences analyzed by MUSCLE3.8.31 (Figure 2). HMG domains showed a higher percentage of identity value among different species than other domains (Figure 1A,B). Moreover, since the HMG domain in the PoSox9a and PoSox9b protein were highly conserved, the genetic and evolutionary diversity in Sox9 among species was caused by the variety of the peptide sequences other than the HMG domain.

#### 2.1.3. Genomic Structure of *Posox9* Genes

**Figure 3:**
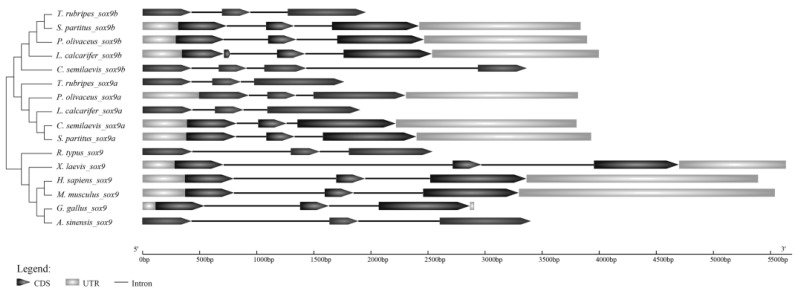
Comparison of genomic organizations of *sox9* between teleosts and tetrapods. Exons are shown in the black wedge, introns are shown in the straight line, and untranslated regions (UTRs) are shown in the gray box. GenBank accession numbers of these sequences are the same as those used in the phylogenetic analysis.

The genomic structures of *sox9* genes in many vertebrates were determined based on their published whole-genome sequences. The *Posox9* genes structures were compared with other vertebrates. As the untranslated region (UTR) sequences of various species were incomplete, we only focused on the open reading frame (ORF) sequences. Analysis of the genomic structure showed that both *Posox9a* and *Posox9b* ORF contained three exons and two introns (Figure 3). Compared with the *sox9* of other species, the genomic structures of the two duplicates of Japanese flounder *sox9* were conserved. All exon–intron boundaries were consistent with the 5′-GT and 3′-AG splicing rule. These results suggested that the two duplicates of Japanese flounder *sox9* genes exhibited a conserved number of exons between flounder species and other tetrapods despite their different gene size.

### 2.2. Relative Expression of Posox9a and Posox9b in Different Tissues

**Figure 4:**
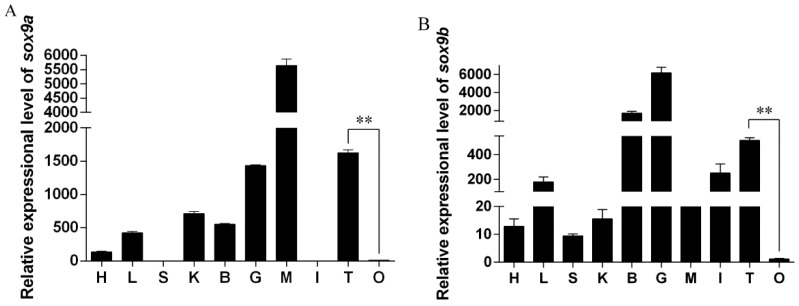
Expression of *Posox9a* and *Posox9b* mRNA in tissues quantified by qRT-PCR. (**A**) *Posox9a*; (**B**) *Posox9b*. Abbreviations: H: heart; L: liver; S: spleen; K: kidney; B: brain; G: gill; M: muscle; I: intestine; T: testis; O: ovary. Data are shown as mean ± Standard Error of Mean (SEM) (*n* = 3). ** indicates statistical significance (*p* < 0.01).

Tissue-specific expression of *Posox9a* and *Posox9b* in adult Japanese flounder was analyzed by qRT-PCR. *Posox9a* gene expressed at higher levels in the muscle, testis and gill, and at lower levels in the liver, kidney and brain; the transcripts were nearly undetectable in the heart, spleen, intestine and ovary (Figure 4A). Notably, the *Posox9a* expression level in the testis was significantly higher than in the ovary. The *Posox9b* gene expressed at higher levels in the brain and gill, and at lower levels in the intestine and testis; the transcripts were nearly undetectable in the heart, liver, spleen, kidney, muscle, intestine and ovary (Figure 4B). Notably, the *Posox9b* gene also expressed at higher levels in the testis than ovary, which was similar to the expression of *Posox9a* in adult gonads.

**Figure 5:**
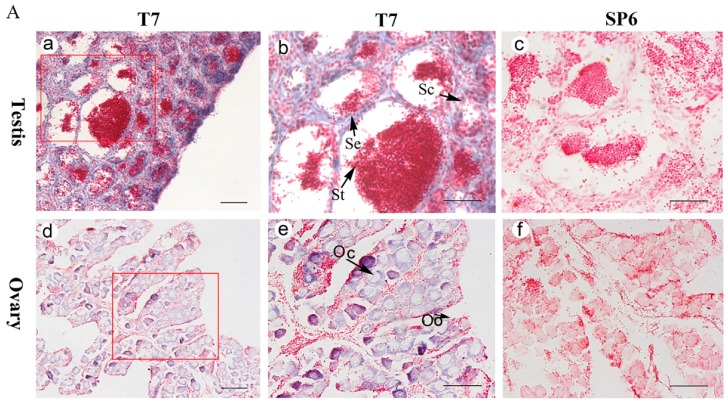

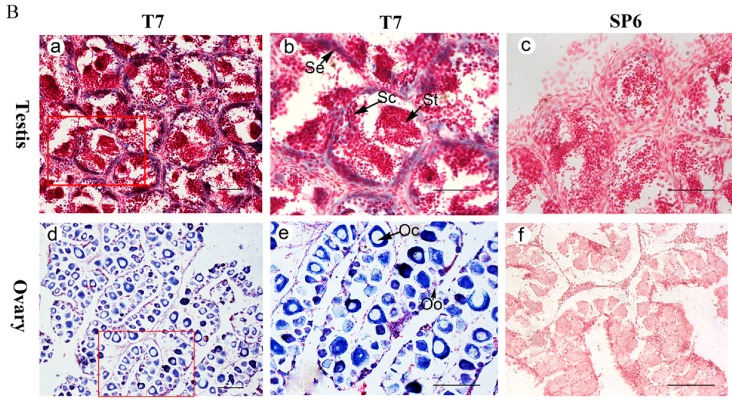
Expression of *Posox9a* and *Posox9b* mRNA in the gonads detected by ISH. (**A**) *Posox9a*; (**B**) *Posox9b*. The positive signals were stained as purple or blue, whereas the negative control with sense probe hybridization was unstained. Abbreviations: Se, Sertoli cells; Sc, spermatocytes; St, spermatid; Oo, oogonia; Oc, oocytes. Scale bars = 100 μm.

The distribution of *Posox9a* and *Posox9b* mRNA in gonadal sections were detected by in situ hybridization (ISH) (Figure 5) using a Digoxin (DIG)-labeled, anti-sense RNA probe. In the adult testis, the cells were mainly comprised of Sertoli cells, spermatocytes and spermatids. ISH results showed that strong positive signals of *Posox9a* and *Posox9b* mRNA were both found in Sertoli cells and spermatocytes (Figure 5A-b,B-b). No signal was detected in spermatids. As for the adult ovary, the cells were mainly comprised of oogonia and oocytes. The signal of *Posox9a* and *Posox9b* mRNA were both detected in the cytoplasm of the oocytes (Figure 5A-e,B-e). No signal was found when detected with the sense probes (Figure 5A-c,f,B-c,f). Spatial expression results showed that *Posox9a* and *Posox9b* mRNA were almost detected in the same location in the gonads. 

### 2.3. Sexually Dimorphic Expression of Posox9a and Posox9b Mrnas in Gonads

**Figure 6:**
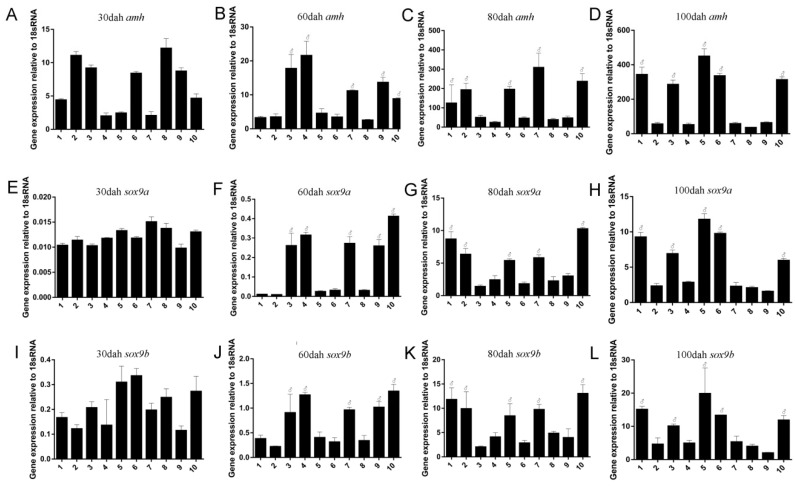
The expression of *sox9a* and *sox9b* genes during gonadal differentiation determined by qRT-PCR analysis. Genetic males and females were distinguished at 30, 60, 80 and 100 days after hatching (dah) according to the expression level of *amh* (**A**–**D**). Numbers 1 to 10 indicate ten individuals. The symbol ♂ indicates genetic males, while the others are genetic females. (**E**–**H**) mRNA levels of *sox9a* in these corresponding genetic male and female gonads from 30 to 100 dah. (**I**–**L**) mRNA levels of *sox9b* in these corresponding genetic male and female gonads from 30 to 100 dah.

Anti-Mullerian hormone (*amh*), also known as Mullerian inhibiting substance (MIS), is responsible for the regression of Mullerian ducts, so it can lead to the masculinization of gonads. It has been reported that Japanese flounder *amh* is sexually dimorphic during sex gonadal differentiation [19]. For the lack of genetic gender marker in Japanese flounder, we referenced the expression of *amh* to distinguish the genetic male and female individuals [20]. The exact expression of *amh*, *Posox9a* and *Posox9b* in gonads during the early development stage (30 to 100 dah) were analyzed by qRT-PCR. At 30 dah, when the gonad was sexually indistinguishable, *amh* RNA mainly expressed in five individuals (Figure 6A, Individual 2, 3, 6, 8 and 9), which were regarded as genetic males, but hardly expressed in the others (Figure 6A, Individual 1, 4, 5, 7 and 10), which were considered as genetic females. Neither *Posox9a* nor *Posox9b* showed obvious sexual dimorphic expression during this stage. After the initiation of sex differentiation (60 dah), higher levels of *amh* mRNA were detected in the five individuals (genetic males) than the others (genetic females) (Figure 6B). The expression levels of both *Posox9a* and *Posox9b* in these genetic males were significantly higher than in genetic females (Figure 6F,J). From 80 to 100 dah, the expression of *amh* mRNA increased remarkably in the gonad of genetic females, but was consistently lower in the genetic males (Figure 6C,D). Similarly, *Posox9a* and *Posox9b* at this stage expressed at higher levels than the stage of 30 dah, with an obvious sexual dimorphic pattern (Figure 6G,H,K,L). It is noteworthy that among all these stages, *Posox9b* expressed at higher levels than *Posox9a*, especially at 30 dah.

### 2.4. Expression Profile of Posox9a and Posox9b after 17α-methyltestosterone Injection

**Figure 7:**
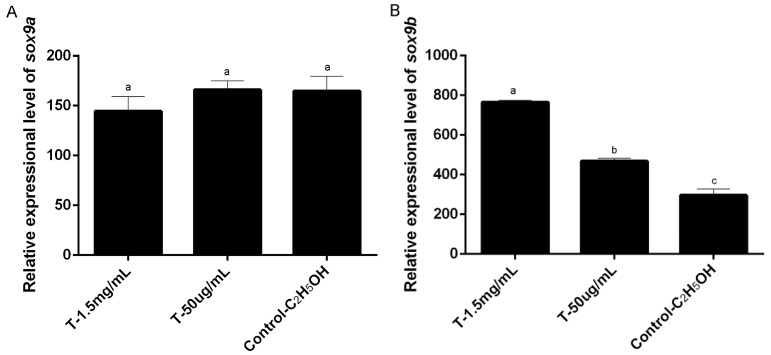
qRT-PCR analysis showing *Posox9a* and *Posox9b* mRNA levels in the testis. (**A**) *Posox9a*; (**B**) *Posox9b.* Data of qRT-PCR were expressed as mean ± SEM (*n* = 3). The same letter used between the groups indicates no significant difference (*p* > 0.05).

After five days of injection of 17α-methyltestosterone, the expression profiles of *Posox9a* and *Posox9b* were studied in adult Japanese flounder by qRT-PCR (Figure 7). The results showed that the expression level of *Posox9b* in the testis increased obviously by 17α-methyltestosterone administration, especially when the 17α-methyltestosterone concentration was 1.5 mg/mL (Figure 7B), whereas *Posox9a* exhibited no significant difference between the experiment groups and control group (Figure 7A).

## 3. Discussion

The *Sox9* gene is an important factor required for cartilage formation and testis determination in mammals [6,7]. In mice, *sox9* is not required for testis cord differentiation after 14 days postcoitum, but is critical for the maintenance of adult testicular functions and fertility [21]. In recent years, more and more research has been performed on the sex determination genes of teleosts. *Sox9* is a candidate gene for the selection of sex determination gene. Japanese flounder, one of the most important species in aquaculture with a stable XX/XY sex determination system, belongs to Pleuronectiformes, Paralichthyidae and *Paralichthys*. Considering its growth differences between male and female individuals and its economic benefits, constructing all-female stocks is of great value. Thus, the exploitation of Japanese flounder sex-related genes has become an important assignment for assisting breeding at the molecular level.

### 3.1. Posox9a and Posox9b Are Highly Conversed in Vertebrates

In this study, two duplicates of the *sox9* gene in Japanese flounder were found, supporting the understanding that *sox9* is duplicated in Japanese flounder, as has also been observed in other fish species, such as zebrafish, fugu and rice field eel due to the whole genome duplication event. Bioinformatic analysis revealed that the genomic structure of *Posox9a* and *Posox9b* were conserved. Evidence gathered from protein sequences and the conserved and characteristic domains demonstrated that *Posox9a* and *Posox9b* encoded Japanese flounder PoSox9a and PoSox9b respectively, and were most closely related to the corresponding homologues of uncovered Sox9 proteins. PoSox9a and PoSox9b, especially the sequences within the conserved HMG-box domain, shared high amino acid sequence identities with other species, implying that PoSox9a and PoSox9b might possess similar regulation and function mechanisms as their homologues.

### 3.2. Both Posox9a and Posox9b Are More Abundant in Testis Than in Ovary

The tissue-specific expression of *Posox9a* and *Posox9b* in adult Japanese flounder was investigated by qRT-PCR to determine whether the genes were uniformly expressed or restricted to specific tissues or genders. The *Posox9a* and *Posox9b* genes were widely expressed in the examined tissues and the expression profiles of the two duplicates were different: the *Posox9a* level was highest in the muscle and *Posox9b* in the gill, indicating they might play different roles in these tissues. Here, we focused on the roles of the two *Posox9* genes in the gonads. The qRT-PCR results showed that the expression levels of *Posox9a* and *Posox9b* were both higher in the testis than in the ovary. The same expression profile was also found in *Anoplopoma fimbria* [14], half-smooth tongue sole (*Cynoglossus semilaevis*) [22] and catfish *sox9a* [23]. Given the sexually dimorphic pattern of *Posox9a* and *Posox9b* expression, it was possible that *Posox9a* and *Posox9b* influenced gonadal development by binding to target genes related to sex determination. This study also found the expression of *Posox9a* in somatic tissues including gill, muscle and kidney and *Posox9b* in the brain and gill, providing further evidence for the idea that the two genes might have an impact on a broad range of biological processes outside the germ line. 

### 3.3. Posox9a and Posox9b Transcripts Are Detected in Sertoli Cells, Spermatocytes and Oocytes

The spatial expression profiles in adult Japanese flounder gonads detected by ISH showed that both *Posox9a* and *Posox9b* expressed in spermatocytes and Sertoli cells in the testis, and oocytes in the ovary. Sertoli cells are primarily under the hormonal regulation of follicle stimulating hormone (FSH) while fulfilling numerous functions geared toward supporting the development and maturation of germinal cells [24]. The importance of Sertoli cells (and FSH) in sperm production has been emphasized by the demonstration that these cells produce a specific protein, the androgen binding protein (ABP), under FSH control [25]. The expression of *Posox9a* and *Posox9b* in Sertoli cells indicated that they might play roles in sperm production. In the present study, the ISH analysis of ovaries showed that *Posox9a* and *Posox9b* transcripts were detected in the cytoplasm of oocytes, indicating that they might also play a role in the process of oogenesis.

### 3.4. Sexually Dimorphic Expression of Posox9a and Posox9b Mrnas in Gonads during Sex Differentiation

It has been found via histological observation that the ovarian and testicular differentiation in *P. olivaceus* juveniles initiated when total length (TL) reached 30 and 37 mm [26,27], thus juveniles of 60 dah (32.7 mm) were selected as the initiation of gonadal differentiation in this study [20]. At this critical point of 60 dah, *Posox9a* and *Posox9a* increased sharply compared with 30 dah, and from then on they both showed obvious sexual dimorphic expression, denoting their important roles in Japanese flounder male sex differentiation. The same expression profiles of *Posox9a* and *Posox9b* in the stage of sex differentiation suggested a possible functional redundancy in these domains, which might facilitate gene function ablation for further evaluation of the roles of *sox9* genes in development and evolution. Another possibility is that there might be a direct interaction between the two *sox9* genes and they work together to promote male sex differentiation, which needs further research for verification.

### 3.5. 17α-methyltestosterone Increased the Expression of Posox9b but Not Posox9a

The sex of Japanese flounder is easily altered by sex steroid hormone treatment during the period of sex determination [28]. 17α-methyltestosterone, produced by Leydig cells and involved in Wolffian duct differentiation [29], is a familiar steroid hormone for inducing the sex-reverse of genetic females to phenotypic males [27,30]. 17α-methyltestosterone treatment during the period from 30–100 dah, which is the critical period of sex determination and differentiation in Japanese flounder [27], causes the masculinization—gonads differentiated to testes. In zebrafish, at a morphological level, 17α-methyltestosterone masculinizes gonads and accelerates spermatogenesis, and these changes are paralleled in the masculinization and de-feminization of gonadal transcriptome. 17α-methyltestosterone treatment can upregulate the expression of the genes involved in male sex determination and differentiation [31]. Thus, in order to explore the roles of *Posox9* genes in gonadal function maintenance, we attempt to find whether 17α-methyltestosterone administration can influence the expression level of the male-related *Posox9* genes of adult Japanese flounder. In our study, five days after 17α-methyltestosterone injection intraperitoneally in 1.5-year-old adults, the expression level of *Posox9b* increased significantly compared with the control group, especially when the 17α-methyltestosterone concentration was 1.5 mg/mL, whereas the change in *Posox9a* was undetectable, manifesting that the effect of androgen on gonadal function maintenance in Japanese flounder might have some relationship with *Posox9b* but not *Posox9a*. Nevertheless, further investigations are needed to determine whether 17α-methyltestosterone directly regulates the expression of the *Posox9b* gene. In addition, the different expression profiles of *Posox9a* and *Posox9b* showed that their functions in gonadal development have already diverged to some degree and the detailed differences need to be further explored. 

## 4. Materials and Methods

### 4.1. Ethics Statement

Animal experiments were all conducted in accordance with the Regulation for the Administration of Affairs Concerning Experimental Animals (China, 1988). The research was also approved by College of Marine Life, Ocean University of China (Qingdao, China).

### 4.2. Fish and Sampling

All larvae and fish were collected from a commercial hatchery in Haiyang, Shandong Province, China. Larvae, cultivated at 18–21 °C, were sampled at 30, 60, 80 and 100 dah. Ten larvae of each stage were sacrificed for RNA extraction and another ten for histological examination and ISH. The average TL was shown in Table 1. The whole abdomen that contained the gonadal anlagen was sampled from juveniles with TL < 50 mm, and gonads were sampled from juveniles with TL > 50 mm.

Six 1.5-year-old adults (three females and three males) were selected randomly. The body length (234–274 mm) and body weight (210–380 g) of those adult individuals were measured. Japanese flounder individuals, anesthetized and sacrificed by severing the spinal cord, were dissected, and the organs, including the heart, liver, spleen, kidney, brain, gill, muscle, intestine and gonad (ovary and testis), were immediately frozen in liquid nitrogen and then stored at −80 °C until RNA extraction. Besides, the remaining gonadal tissues were collected and stored in methanol for ISH. Genders were identified through morphological observation.

The 1.5-year-old male Japanese flounder individuals (*n* = 9), injected with androgen (17α-methyltestosterone) intraperitoneally in concentration of 1.5 mg/mL and 50 μg/mL, were dissected on the fifth day post injection and the testes were immediately frozen in liquid nitrogen and then stored at −80 °C until RNA extraction. The control group was injected with an equivalent dosage of ethanol.

### 4.3. RNA Extraction and cDNA Synthesis

RNA were extracted with Trizol Reagent (Invitrogen, Carlsbad, CA, USA) according to the manufacturer’s protocol, and then treated with RNase-free DNase I (TaKaRa, Dalian, China) to eliminate genomic DNA contamination. After that, 1.5% agarose gel electrophoresis and spectrophotometry was used to detect the concentration and integrity. cDNA was synthesized with total RNA and random hexamer primers using a Reverse Transcriptase M-MLV Kit (TaKaRa) following the manufacturer’s protocol and verified by PCR using β-actin gene specific primers (Table 2) which spanned different exons.

### 4.4. Molecular Cloning of Posox9a and Posox9b

Through searching the *de novo* transcriptome sequencing data of Japanese flounder [32], we found two different sequences of Japanese flounder *sox9* named *Posox9a* and *Posox9*b, respectively. Then, two pairs of primers (*Posox9a*-Fw/Rv; *Posox9b*-Fw/Rv, Table 2) were designed to confirm the core fragments and ensure sequence accuracy. The total RNA used for obtaining *Posox9a* and *Posox9b* cDNA was extracted from adult testis tissue. First-strand cDNA was synthesized from 1 μg of total RNA using M-MLV reverse transcriptase (RNase H-^−^) (TaKaRa) and random primers. The amplified PCR products of the appropriate size were separated by agarose gel electrophoresis, purified using the Zymoclean Gel DNA Recovery kit (Zymo Research, Orange, CA, USA), and cloned into a pMD-19T Vector (TaKaRa) for sequencing.

### 4.5. Sequence Analysis

Sequence data were assembled and analyzed using software suite Lasergene v7.0 (DNASTAR, Madison, WI, USA). Homologous nucleotide and protein sequences were confirmed through using the BLASTn and BLASTx search algorithm in NCBI (http://www.ncbi.nlm.gov/blast). Multiple alignments of amino acid sequences were performed using MUSCLE and refined by GBLOCKS. A phylogenetic tree was generated using maximum likelihood algorithm by MEGA6.0 with a WAG model based on the amino acid sequences analyzed by MUSCLE. 

### 4.6. Quantitative Real-Time PCR (qRT-PCR)

The cDNA templates used for qRT-PCR analysis were generated using the method described above and further amplified with β-actin primers to exclude any possible residual DNA contamination. Primers of *Posox9a* and *Posox9b* for qRT-PCR were designed outside the conserved domains to prevent any non-specific amplification. qRT-PCR was performed in a 20 μL system containing 10 ng template cDNA, SYBR qPCR SuperMix (Novoprotein, Shanghai, China), 2.5 μmol/L each of specific forward and reverse primers and RNA-free water. *Posox9a*, *Posox9b* and *amh* amplicons were separated through 1.5% agarose gel electrophoresis, purified using the Zymoclean Gel DNA Recovery Kit, cloned into the pMD-19T Vector. The positive clone was verified by sequencing and used for plasmid construction. A standard curve and amplification efficiency were derived from the serial dilutions of the relative plasmid. Melting curves were generated at the end of each run to assess the specificity of the amplicons and the absence of dimers. A negative control (no template) was always included. The result of absolute quantification for the target genes were calculated by the standard curve method with 18*S* RNA as the reference gene. All qRT-PCR assays for a particular gene were conducted under identical conditions in triplicate. All primers were listed in Table 2.

### 4.7. ISH

ISH of *Posox9a* and *Posox9b* expression in the gonads was performed using a 393-bp and 459-bp probe spanning the ORF of cDNA, which was amplified by specific primers (Table 2). DIG-labeled RNA sense and anti-sense probes were synthesized using the DIG RNA Labeling Kit (SP6/T7) (Roche, Mannheim, Germany) according to the manufacturer’s instructions. ISH on paraffin sections of the gonads was performed according to a previous study [33].

### 4.8. Statistical Analysis

Results were expressed as mean ± standard error of the mean. qRT-PCR data were analyzed using one-way analysis of variance followed by least significant difference test using SPSS 20.0 (IBM, New York, NY, USA). Significance was set at *p* value < 0.05.

## 5. Conclusions

In this study, we found two duplicates of *sox9* in Japanese flounder (*Paralichthys olivaceus*) named *Posox9a* and *Posox9b,* respectively. The two genes were conserved in terms of phylogenetic and gene structure analyses. In adult gonads, the expression levels of *Posox9a* and *Posox9b* were both higher in the testis than the ovary, and their mRNA were both detected in the cytoplasm of oocytes, Sertoli cells and spermatocytes. During sex differentiation, the expression level of *Posox9a* and *Posox9b* were both higher in male-preferred individuals than female-preferred individuals. 17α-methyltestosterone injection upregulated the expression of *Posox9b*, but had no influence on *Posox9a* in testis. These results suggested that Japanese flounder *Posox9a* and *Posox9b* might perform an essential function in gonadal development. Our study provides a foundation for further exploration of the roles of *sox9* genes during the sex determination and differentiation, spermatogenesis and gonadal function maintenance of Japanese flounder.

## Figures and Tables

**Table 1:** The average total length (TL) of sampled *P. olivaceus* larvae.

Period	30 Dah	40 Dah	50 Dah	60 Dah	70 Dah	80 Dah	90 Dah	100 Dah
TL (mm)	11.4	16.6	24.1	32.7	43.6	50.4	54.9	61.4

**Table 2:** Primers used in this study.

Primer Name	Sequence (5′–3′)	Usage
Posox9a-Fw	ATGAATCTCCTCGACCCTTACC	ORF amplification
Posox9a-Rv	TCAGGGTCTGGTGAGCTGG	ORF amplification
Posox9a-qRT-Fw	AACGAAGGCGAGAAGCGG	qRT-PCR
Posox9a-qRT-Rv	ATGGCATTAGGAGAAATGTGCG	qRT-PCR
Posox9a-ISH-Fw	AATCCTCTGTGAGGACTTATTG	ISH probe
Posox9a-ISH-Rv	CAGATTCCGTCTCTCTTTCTC	ISH probe
Posox9b-Fw	ATCTGTGATACGCTGTTCTTT	ORF amplification
Posox9b-Rv	CTCTTCACGGCCTGGAC	ORF amplification
Posox9b-qRT-Fw	GCGAGCAAACGCACATA	qRT-PCR
Posox9b-qRT-Rv	CCAAAGTCGATGTTGAGCTG	qRT-PCR
Posox9b-ISH-Fw	CTTTGAATTGGCTCACAACAG	ISH probe
Posox9b-ISH-Rv	ACTGATGACACGGTCATATC	ISH probe
amh-qRT-FW	AGCACTGACAGTTTCTCATCC	qRT-PCR
amh-qRT-RV	GTAAGACTGATCCCGATGAACTG	qRT-PCR
18s-qRT-FW	GGTAACGGGGAATCAGGGT	qRT-PCR
18s-qRT-RV	TGCCTTCCTTGGATGTGGT	qRT-PCR
β-actin-qRT-Fw	GAGATGAAGCCCAGAGCAAGAG	qRT-PCR
β-actin-qRT-Rv	CAGCTGTGGTGGTGAAGGAGTAG	qRT-PCR
